# A surgical case of radiotherapy induced esophageal perforation accompanying pyogenic spondylodiscitis: a case report

**DOI:** 10.1186/s40792-017-0368-1

**Published:** 2017-08-31

**Authors:** Shuntaro Yoshimura, Kazuhiko Mori, Koichiro Kawasaki, Asami Tanabe, Susumu Aikou, Koichi Yagi, Masato Nishida, Hiroharu Yamashita, Sachiyo Nomura, Masayoshi Fukushima, Hideomi Yamashita, Yasuhiro Yamauchi, Yasuyuki Seto

**Affiliations:** 1 Department of Gastrointestinal Surgery, Graduate School of Medicine, University of Tokyo, Hongo 7-3-1, Bunkyo-ku, Tokyo, 113-8655 Japan; 2 Department of Gastrointestinal Surgery, Mitsui Memorial Hospital, Kanda-Izumi-cho 1, Chiyoda-ku, Tokyo, 101-8643 Japan; 3 Department of Orthopaedic Surgery, University of Tokyo, Hongo 7-3-1, Bunkyo-ku, Tokyo, 113-8655 Japan; 4 Department of Radiation Oncology, University of Tokyo, Hongo 7-3-1, Bunkyo-ku, Tokyo, 113-8655 Japan; 5 Department of Respiratory Medicine, University of Tokyo, Hongo 7-3-1, Bunkyo-ku, Tokyo, 113-8655 Japan

**Keywords:** Stereotactic body radiotherapy, Esophageal perforation, Spondylodiscitis, Nontransthoracic esophagectomy

## Abstract

**Background:**

Stereotactic body radiotherapy has been a treatment choice for lung cancer, especially in medically inoperable patients. However, the acute and late toxicity to adjacent organs have been reported as an uncommon but severe adverse effect.

**Case presentation:**

A 65-year-old male was presented with his back pain and pyrexia. He had been followed up for non-small-cell lung cancer, which was treated by the stereotactic body radiotherapy 4 years prior to the current visit. The endoscopy revealed an esophageal perforation on its left side in the upper thoracic locus. Because of his poor lung function, he was managed by the conservative treatment. After 3 months, his back pain recurred with developing paraplegia in the lower extremities. The MRI revealed an abscess formation at the posterior side of the upper thoracic esophagus which destroyed the vertebral body and compressed the spinal cord. Laminectomy and two-stage operation—the first stage, nontransthoracic esophagectomy, cervical and transhiatal approach using mediastinoscope and laparoscope, and the second stage, esophageal reconstruction—were performed.

**Conclusion:**

This complex disease status was successfully managed by the orthopedic surgery followed by a two-stage esophagectomy without transthoracic approach.

## Background

Stereotactic body radiotherapy (SBRT) has been a treatment choice for lung cancer, especially in medically inoperable patients, and is indicated with a definitive intention. However, the acute and late toxicity to adjacent organs, such as the bronchi, the aorta, the brachial plexus, and the esophagus, have been reported as an uncommon but severe adverse effect.

## Case presentation

A 65-year-old male was presented with his persistent back pain and daily pyrexia. He had suffered from chronic dyspnea on effort as a symptom of the chronic obstructive pulmonary disease and used home oxygen therapy of 2 l/min on occasional use. He had been followed up for non-small-cell lung cancer (NSCLC) of the left upper lobe (T2aN1M0), which was treated by the SBRT (50 Gy in four fractions) 4 years prior to the current visit. SBRT was effective enough to achieve complete response of the disease, and the patient had developed no evident recurrent disease so far. After the completion of SBRT, he occasionally complained about postprandial soreness in the upper chest for 4 years until the evaluation by the following clinical examinations.

**Fig. 1:**
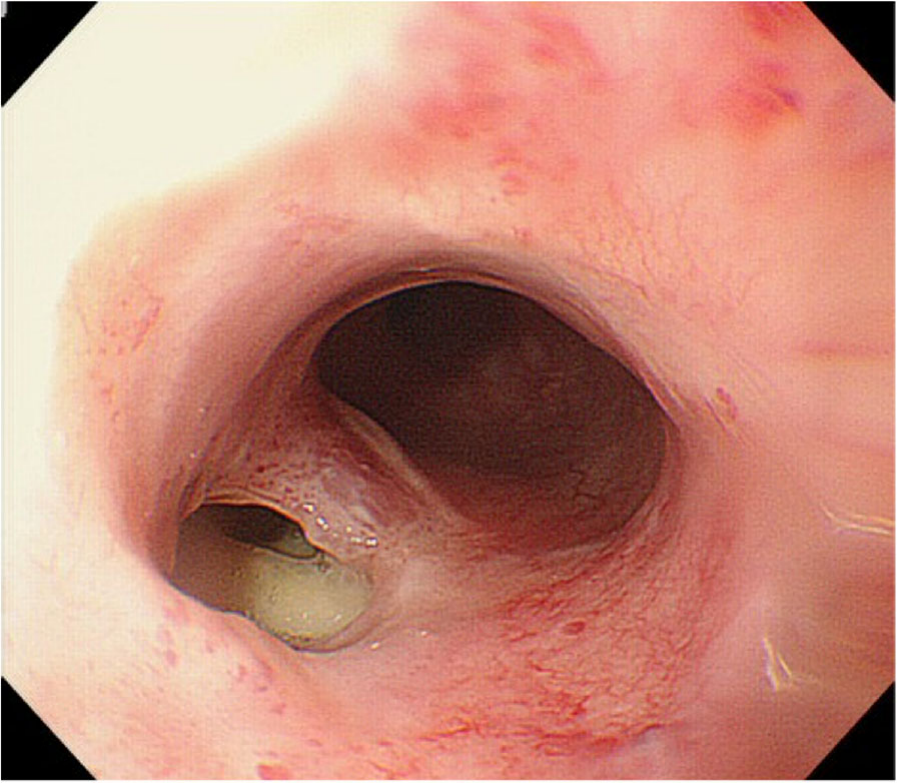
Endoscopy revealed an esophageal perforation in the left side of the upper thoracic esophagus

The blood examination revealed marked leukocytosis and elevated level of C reactive protein (CRP) ( Table 1 ). A computed tomography (CT) revealed thickening of the pleura and the soft tissue adjacent to the left side of the upper thoracic esophagus. The endoscopy revealed an esophageal perforation on its left side in the upper thoracic locus ( Fig. 1 ). With a diagnosis of the esophageal perforation and mediastinitis, he was referred to our department.

**Table 1:** Blood examination on admission

WBC	13,200/μl	AST	16 U/L
RBC	445 × 10 ^4^ /μl	ALT	18 U/L
Hb	13.8 g/dl	BUN	17.0 mg/dl
Ht	41.90%	Cre	1.03 mg/dl
Plt	39.8 × 104/μl	Na	140 mEq/L
CRP	29.9 mg/dl	K	3.6 mEq/L

### Primary treatment

Since his lung function was quite poor with a vital capacity of 2.31 l and a forced expiratory volume per one second (FEV 1.0) of 0.52 l, a conservative policy was favored. Enterostomy was established by a surgical operation, and he was followed up with a long-term prohibition of oral intake under the enteral nutrition. The inflammatory markers such as leukocyte count and CRP showed improvement, and the patient was discharged to be followed up by the regular surveillance by blood tests and CT.

### Onset of the vertebral osteomyelitis and an emergent operation

**Fig. 2:**
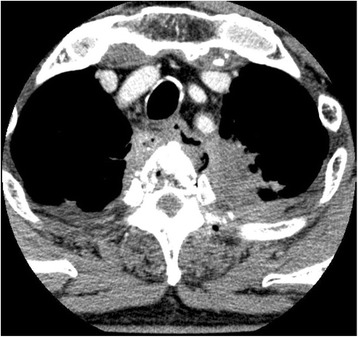
Contrast-enhanced chest CT scan suggested the perforation of the esophagus in the upper left lateral wall with fluid and air in the posterior mediastinum. Osteolytic changes with free air space were observed within the vertebral body

**Fig. 3:**
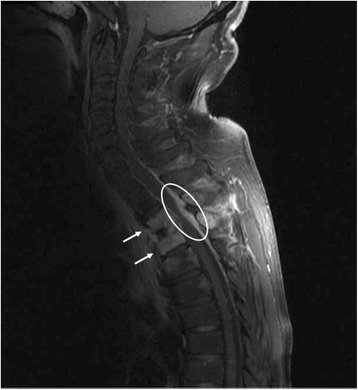
MRI at the onset of paraplegia showed the destructions of the Th2–3 vertebral bodies (arrow) and the intervertebral disc accompanying epidural abscess (circled high intensity area) compressing the spinal cord at the level of Th2–3

**Fig. 4:**
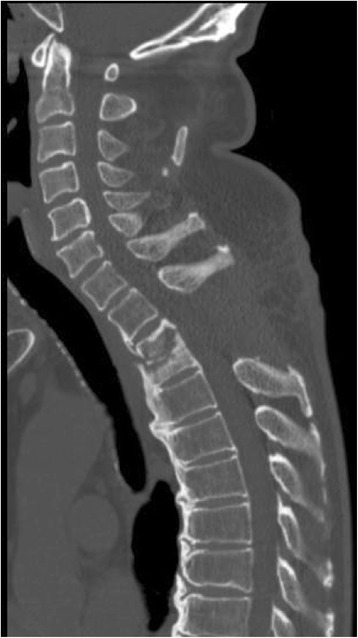
Laminectomy of Th2–3 was performed. Osteolytic change was observed in the Th2–3 vertebral bodies

After 3 months, his back pain recurred with a sudden and steep worsening and he revisited our department on an emergency occasion. The patient developed paraplegia with muscle weakness in the lower extremities. An emergent CT demonstrated a spread of the periesophageal abscess which invaded and destroyed the vertebral body ( Fig. 2 ). The condition was emergently consulted to an orthopedician. The magnetic resonance imaging (MRI) revealed an abscess formation at the posterior side of the upper thoracic esophagus which penetrated and destroyed the intervertebral disc and vertebral body and compressed the spinal cord at the level of Th2–3 ( Fig. 3 ). His condition was considered as an indication of emergent surgery for an acute spinal cord injury, and the laminectomy of the Th2–3 and debridement of the malgranulation were performed ( Fig. 4 ). *Citrobacter koseri* and *Streptococcus mitis* were isolated from the specimen of an epidural abscess. After the surgery, his neurologic symptom had gradually improved.

### Surgery for esophageal perforation

**Fig. 5:**
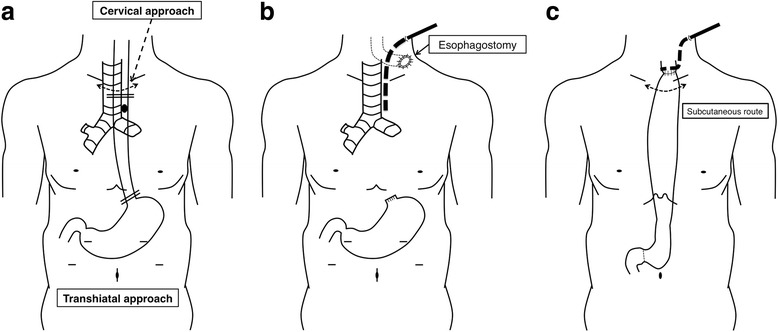
Schematic illustrations of the two-stage operation. **a** Subtotal esophagectomy was performed in the first stage operation with the combination of transcervical and transhiatal approaches. Dashed arrow, cervical skin incision; double lines, oral and anal margins of the esophagectomy; black dot, the location of the esophageal perforation. **b** Status after the first stage operation. Cervical esophagostomy was placed in the left side of the patient’s neck and a 19-Fr drainage tube (dotted thick line) was placed via the left side of the patient’s neck into the upper mediastinum. **c** Status after the second stage operation. Gastric conduit was lifted via a subcutaneous route, and an esophago-gastric anastomosis was performed. A 19-Fr drainage tube (dotted thick line) was placed behind the anastomosis via the left side of the patient’s neck

The first of the two-stage operation was performed 8 days after laminectomy: the first stage, nontransthoracic esophagectomy, cervical and transhiatal approach using mediastinoscope and laparoscope, described below ( Fig. 5 a), and the second stage, esophageal reconstruction.

In the first stage, the upper mediastinal manipulation was performed by a cervical approach via a collar incision and with mediastinoscopic guidance. There were firm adhesions between the dorsal side of the esophagus and the anterior vertebral ligament presumably caused by the esophagus perforation. After the dissections of the esophagus from the bilateral pleura, dissection of the dorsal side of the esophagus was performed and the perforated site of the upper thoracic esophagus was confirmed in the left-dorsal aspect of the esophageal wall. Subsequently, dissections between the left side wall of the trachea and the esophagus and between the anterior mediastinum and the esophagus were carried out. The left recurrent nerve was sharply dissected from the esophagus and preserved. After these transcervical procedures assisted by the mediastinoscope, the upper thoracic esophagus including the perforated site was free from attachments to the adjacent anatomical structures.

Following to the abovementioned upper mediastinal manipulations, transhiatal approach for the middle and lower mediastinum was performed laparoscopically. Firstly, the anterior side of the esophagophrenic ligament was incised and the mediastinum was entered. The lower esophagus was dissected from the bilateral pleura, the pericardia, and the aorta. As the dissection proceeded to the cranial side beside the esophagus, the dissection was reached to the hollow space surrounding the upper thoracic esophagus which was created by the prior dissections via a cervical approach. Consequently, the whole thoracic esophagus was ready to harvest. After the esophago-gastric junction was transected, the mobilized esophagus was pulled up from the cervical incision. Finally, a cervical esophagostomy was placed in the left side of his neck and a 19-Fr drainage tube was inserted via the left side of his neck into the upper mediastinum (Fig. 5 b).

In the second stage, surgery for reconstruction was performed 4 weeks after the first stage surgery (Fig. 5 c). In the reconstruction surgery, gastric conduit was created with linear staplers via upper median laparotomy. The conduit was lifted via a subcutaneous route, and an esophago-gastric hand-sewn anastomosis (a single layer of Gambee sutures) was performed.

### Postoperative course

Although left recurrent nerve palsy and anastomotic stricture occurred, the patient’s condition gradually improved with conservative treatment (swallowing rehabilitation and endoscopic bougienage) and he was discharged from hospital at 183 days after esophagectomy. His follow-up was discontinued 12 months after the discharge because of the acute myeloid leukemia which was cared by the best supportive care. By the time, he had been free from any signs of local recurrence or neurological impairments and able to take meals enough to abandon the enteral nutrition.

### Discussion

Surgical resection is regarded as a standard treatment for early NSCLC while radiotherapy has been offered to patients who are not suitable for surgery due to medically inoperable factors such as poor pulmonary function or severe cardiovascular dysfunction [1]. SBRT is currently defined as a technique for delivering external beam radiotherapy with a high degree of accuracy to an extra-cranial target, using high doses per fraction, and now considered as the first-line treatment option for medically inoperable patients affected by early NSCLC [2]. For peripheral lung tumors, low treatment-related toxicity rates have been confirmed in prospective trials, as these tumors are mainly surrounded by lung tissue [3]. On the other hand, SBRT for central located tumors (< 2 cm from the main bronchus) has a potential risk of developing severe, potentially life-threatening toxicities because of the proximity of the target field to critical organs such as the main bronchus, the esophagus, and the heart [4]. When SBRT is employed to treat centrally located NSCLCs, the esophagus is typically one of the most crucial organs at risk owing to its close proximity to the radiation field. The adverse effects on the esophagus are well-known complications of SBRT for centrally located lung tumors. These esophageal complications range from esophagitis to esophagus ulcer resulting in stricture, perforation, and/or tracheo-esophageal fistula, and the frequency of grade 1–2 and over grade 3 toxicity according to the Common Terminology Criteria for Adverse Events has been reported as up to 12.8% and up to 6.8%, respectively [5]. Late esophagus toxicities as defined to occur more than 90 days are usually emerging 3 and 18 months after the termination of radiotherapy [5]. Sainathan et al. reported two cases of delayed esophageal perforation after SBRT for locally recurrent NSCLC [6]. In these two cases, esophageal perforation occurred 5 and 7 months after SBRT for centrally located recurrent tumors. As for the case in this report, the first presentation of the esophageal fistula was 4 years after the SBRT and target NSCLC was located in the left pulmonary apex (more than 2 cm distant from the main bronchus). Moreover, to our knowledge, this is the first case report of SBRT-induced esophageal perforation form periesophageal abscess, and this abscess invaded the vertebral body through the intervertebral disc and developed paraplegia.

Several managements for esophageal perforation have been reported such as conservative treatment, esophagectomy, endoscopic clipping, and endoscopic placement of stent [7, 8] as two cases were treated by a covered esophageal stent [6]. In the current case, esophageal perforation occurred in the irradiated esophagus, which might be too friable and vulnerable to tolerate the placement of metal stent. There are several case reports that a covered self-expandable and retrievable stent (HANAROSTENT) was effective and successfully manage the esophago-gastric anastomosis fistula [9, 10]. Although HANAROSTENT could have been a possible choice in this case, we considered that the separation of the gastrointestinal contents from the severely infected mediastinum would be the most reliable measure to manage this critical vertebral infection. Nonetheless, the patient’s severe pulmonary dysfunction (vital capacity of 2.31 l and FEV 1.0 of 0.52 l) made the transthoracic esophagectomy an unsuitable choice. Therefore, we consider the nontransthoracic esophagectomy and delayed reconstruction as a necessary and adequate procedure for this complicated esophageal perforation. The upper mediastinal anatomical structures adjacent to the esophagus may be susceptible to surgical injuries owing to the previous radiotherapy, periesophageal abscess formation, and adhesive changes. Therefore, conventional transhiatal esophagectomy would not be a suitable choice because it includes blind and blunt dissections. The use of mediastinoscope was advantageous to confirm the surgical view directly and prevented secondary injuries on the anatomical structures adjacent to the esophagus.

## Conclusions

In summary, we present a case of SBRT-induced esophageal perforation with mediastinal abscess penetrating the vertebral disc and presenting paraplegia. This complex disease status was successfully managed by the orthopedic surgery followed by a two-stage esophagectomy without transthoracic approach.

## Abbreviations

CRP: C reactive protein
CT: Computed tomography
MRI: Magnetic resonance imaging
NSCLC: Non-small-cell lung cancer
SBRT: Stereotactic body radiotherapy
